# Is age an advantage? Improved outcomes after medial unicompartmental knee arthroplasty in patients aged ≥70 years

**DOI:** 10.1002/jeo2.70479

**Published:** 2025-11-04

**Authors:** Miguel Ángel Muñoz‐Sánchez, Ana Martínez‐Crespo, Carmen Tara‐Abad, María Ríos‐Morón, Elvira Montañez‐Heredia, Juan Miguel Gómez‐Palomo

**Affiliations:** ^1^ Department of Orthopedic Surgery and Traumatology Virgen de la Victoria University Hospital Málaga Spain; ^2^ Biomedical Research Institute of Málaga (IBIMA) Málaga Spain; ^3^ Department of Orthopedic Surgery and Traumatology Vithas Xanit International Hospital Málaga Spain

**Keywords:** elderly patients, functional outcomes, medial compartment osteoarthritis, perioperative complications, postoperative pain, unicompartmental knee arthroplasty

## Abstract

**Purpose:**

To determine whether patients aged ≥ 70 years undergoing medial unicompartmental knee arthroplasty achieve superior clinical outcomes compared with younger patients, addressing the current lack of age‐specific evidence in this setting.

**Methods:**

A retrospective cohort study was conducted, and 1:1 propensity‐score matching was employed using the following variables: age, body mass index, Charlson Comorbidity Index, baseline Knee Society Score (KSS), and preoperative visual analogue scale (VAS) for pain. The matched cohort included 76 patients (38 elderly patients aged ≥70 years and 38 younger patients <70 years), all with a minimum of 12 months' follow‐up. Primary endpoints were postoperative VAS pain, KSS, and perioperative complications.

**Results:**

In the matched cohort, elderly patients had lower postoperative pain (VAS 2.1 ± 2.2 vs. 3.1 ± 2.4; *p* = 0.037) and higher KSS (82.5 ± 14.5 vs. 73.4 ± 16.9; *p* = 0.021) than younger patients, while also reporting a higher rate of postoperative satisfaction (97.4% vs. 84.2%; *p* = 0.049) and fewer complications (2.6% vs. 13.2%; *p* = 0.048), at a minimum of 12 months' follow‐up.

**Conclusion:**

In appropriately selected candidates, patients aged ≥ 70 years undergoing medial unicompartmental knee arthroplasty achieve clinical outcomes and satisfaction that are at least comparable—and in several measures superior—to those of younger patients at ≥12 months, supporting age ≥70 years as a viable indication.

**Level of Evidence:**

Level III, retrospective comparative study.

## Graphical abstract

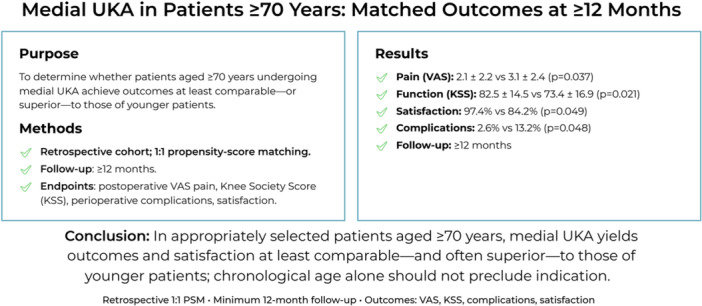

**Abbreviations**
BMI: body mass index
EQ‐5D: EuroQol‐5 Dimension
HSS: hospital for special surgery
KSS: Knee Society Score
ROM: range of motion
UKA: unicompartmental knee arthroplasty
VAS: Visual Analogue Scale

## INTRODUCTION

Unicompartmental knee arthroplasty (UKA) is an established treatment for isolated medial compartment osteoarthritis, offering rapid recovery, less soft‐tissue disruption, and preservation of native knee kinematics [16, 18]. Recent evidence shows excellent outcomes and survivorship even in younger candidates, challenging historical age cut‐offs [10, 11], and is consistent with recent European series reporting contemporary indications and outcomes in medial UKA [13]. Over the past decade, indications have evolved; age, body mass index (BMI), or activity level are increasingly considered relative rather than absolute contraindications when alignment, ligament stability and compartmental degeneration are appropriate [16]. Surgical expertise and surgeon/institutional volume are strong determinants of outcomes and survivorship [1]. Contemporary literature further underscores that implantation technique and enabling technologies can influence alignment accuracy, outlier rates and patient‐reported outcomes [4, 6, 19].

Despite these advances, few studies have specifically evaluated outcomes in patients aged ≥ 70 years, and available cohorts are often limited or heterogeneous [2, 5, 8, 21]. Emerging data further suggest that alignment‐preserving, ligament‐guided approaches may enhance functional results after UKA [20], and their integration with enabling technologies has been associated with fewer outliers, favourable survivorship and improved patient‐reported outcomes [6, 19]. Accordingly, the objective of this study was to compare pain, function, complications, and satisfaction after medial UKA between patients aged ≥ 70 and < 70 years at a minimum of 12 months' follow‐up. It was hypothesised that chronological age ≥ 70 years would not, by itself, preclude safe and effective UKA.

## MATERIALS AND METHODS

### Study design and ethical approval

A retrospective cohort study was employed to evaluate the clinical outcomes of medial unicompartmental knee arthroplasty (UKA) in elderly patients ( ≥ 70 years) with isolated medial compartment osteoarthritis. The study protocol was approved by the Research Ethics Committee of the Province of Málaga (reference SICEIA‐2024‐001425). Written informed consent was obtained from all participants or their legally authorised representatives prior to inclusion.

The study adhered to the ethical principles outlined in the Declaration of Helsinki, as revised in Fortaleza, Brazil, in 2013. All data were collected, stored, and analysed in accordance with institutional guidelines for confidentiality and data protection. No a priori sample‐size calculation was performed; all eligible cases during the study period were included, and the primary analysis was confined to the propensity‐score matched (PSM) cohort.

### Patient selection

This retrospective study was conducted using data from a prospectively maintained institutional surgical database. All patients who underwent medial UKA between January 2017 and January 2024 were retrospectively screened for eligibility based on predefined inclusion and exclusion criteria. The indication for surgery in all cases was symptomatic, functionally limiting medial unicompartmental osteoarthritis unresponsive to conservative measures, including pharmacologic management, physical therapy, and intra‐articular injections.

**Inclusion criteria were:**

- Age ≥ 50 years at the time of surgery (this broader age criterion allowed for the inclusion of a younger comparative cohort [<70 years], essential to specifically evaluate the influence of advanced age [≥70 years]).
- Diagnosis of isolated medial compartment osteoarthritis (Kellgren–Lawrence Grade III–IV or Ahlbäck Grade 2–4).
- Undergoing cemented mobile‐bearing medial UKA.
- Availability of a minimum clinical follow‐up of 12 months postoperatively.

**Exclusion criteria were:**

- Prior surgery on the same knee (e.g., high tibial osteotomy, ligament reconstruction).
- Radiographic evidence of lateral or patellofemoral osteoarthritis classified as Ahlbäck Grade ≥ 3.
- Inflammatory arthritis or active joint infection.
- Severe coronal deformity (>10° varus or >5° valgus), flexion contracture >10°, or ligamentous instability.
- Patient relocation or inability to attend follow‐up evaluations.
- Refusal to participate in the study or withdrawal of consent.

Based on age at the time of surgery, patients were divided into two cohorts:

- Elderly group: patients aged 70 years or older (range 70–86 years).
- Younger group: patients younger than 70 years (range 51–69 years).

All patients meeting the criteria were included consecutively and treated following a standardised surgical and rehabilitation protocol. All inferential comparisons reported in this manuscript are restricted to the PSM cohort (38 ≥ 70 years; 38 < 70 years); unmatched data were used solely to construct the matching set.

### Radiological assessment

Preoperative imaging. All patients underwent weight‐bearing anteroposterior (AP) and lateral knee radiographs, axial patellofemoral views (Ficat method), and standing full‐length hip–knee–ankle (HKA) radiographs. The HKA angle was defined as the angle between the mechanical femoral axis (centre of femoral head to intercondylar notch) and the mechanical tibial axis (centre of tibial spines to ankle centre); by study convention, positive values indicate varus and negative values indicate valgus. Acquisition followed a standardised positioning protocol (patellae forward, full extension, calibrated source‐to‐image distance). MRI was obtained when needed to confirm cruciate ligament integrity and to exclude advanced lateral or patellofemoral degeneration. Postoperative imaging and measurements. Routine follow‐up included weight‐bearing AP and lateral knee radiographs. We recorded overall femorotibial alignment (HKA, °) and component positioning on standardised weight‐bearing views:

- Femoral component—coronal angle (°): angle between the distal femoral component and the mechanical femoral axis (neutral ≈ 90°).
- Tibial component—coronal angle (°): angle between the tibial baseplate and the mechanical tibial axis (neutral ≈ 90°).
- Femoral component—sagittal flexion (°): angle between the component's sagittal axis and the femoral anatomical axis (flexion positive).
- Tibial component—posterior slope (°): angle between the tibial baseplate and a line perpendicular to the tibial mechanical axis (posterior positive).

Measurements were performed on calibrated digital images within the institutional PACS using standard goniometric tools. Two fellowship‐trained knee surgeons measured all images independently, blinded to group allocation and outcomes; a random 20% subset was re‐measured after ≥ 2 weeks. Table 1, 2, 3 summarise the variables and measurements reported in the Results.

**Table 1:** Baseline characteristics of the propensity‐matched cohort.

Variable	≥70 years (PSM *n* = 38)	<70 years (PSM *n* = 38)	*p* value	Effect size
Sex — Male, *n* (%)	17 (44.7%)	18 (47.4%)	0.83^a^	0.03^b^
Sex — Female, *n* (%)	21 (55.3%)	20 (52.6%)	0.83^a^	—
BMI (kg/m²), mean ± SD	30.4 ± 2.9	30.1 ± 3.0	0.62*	0.10^c^
Charlson Comorbidity Index ≥1, *n* (%)	16 (42.1%)	15 (39.5%)	0.82^a^	0.03^b^
Ahlbäck grade (femorotibial), mode	2	2	—^d^	—
Ahlbäck grade (patellofemoral), mode	2	2	—^d^	—
HKA (°), mean ± SD	6.1 ± 2.1	6.0 ± 2.2	0.84*	0.05^c^

Abbreviations: BMI, body mass index; HKA, hip–knee–ankle; PSM, propensity‐score matched; SD, standard deviation.

* Mann–Whitney *U* test (two‐sided) for continuous variables.

^a^ Chi‐square or Fisher's exact test (two‐sided) for categorical variables, as appropriate.

^b^ Effect size for categorical distribution computed as Cramér's V.

^c^ Effect size for continuous variables computed as Cohen's *d* (pooled SD).

^d^ Modes are descriptive; no category‐wise contrast was performed in this table.

**Table 2:** Primary and key secondary postoperative outcomes in the propensity‐matched cohort.

Outcome	≥70 years (*n* = 38)	<70 years (*n* = 38)	*p* value	Effect size
VAS (postoperative)	2.1 ± 2.2	3.1 ± 2.4	0.037*	–0.45^a^
ΔVAS (post – pre)	–5.4 ± 2.5	–4.3 ± 2.7	0.019*	–0.41^a^
KSS (postoperative)	82.5 ± 14.5	73.4 ± 16.9	0.021*	0.60^a^
ΔKSS (post – pre)	35.0 ± 13.8	27.0 ± 17.1	0.026*	0.52^a^
Complications, *n*/*N* (%)	1/38 (2.6%)	5/38 (13.2%)	0.048^b^	—
“Satisfied”, *n*/*N* (%)	37/38 (97.4%)	32/38 (84.2%)	0.049^b^	0.25^c^

Abbreviations: KSS, Knee Society Score; SD, standard deviation; VAS, visual analogue scale.

* Mann–Whitney *U* test (two‐sided) for continuous outcomes.

^a^ Effect size for continuous variables computed as Cohen's *d* (pooled SD).

^b^ Chi‐square or Fisher's exact test (two‐sided) for categorical outcomes, as appropriate.

^c^ Effect size for categorical distribution computed as Cramér's V.

**Table 3:** Radiographic outcomes and component positioning in the propensity‐matched cohort.

Variable	≥70 years (PSM *n* = 38)	<70 years (PSM *n* = 38)	*p* value	Effect size
HKA angle (°; positive = varus)	4.2 ± 2.5	4.1 ± 2.7	0.840*	0.04^a^
Femoral component coronal alignment (°)	5.8 ± 3.3	5.6 ± 3.2	0.740*	0.06^a^
Tibial component coronal alignment (°)	3.0 ± 1.7	2.9 ± 1.6	0.720*	0.06^a^
Femoral component sagittal flexion (°; flexion positive)	4.0 ± 2.0	3.7 ± 2.1	0.470*	0.15^a^
Tibial component posterior slope (°; posterior positive)	3.8 ± 2.0	3.6 ± 2.0	0.680*	0.10^a^

*Note*: Angles measured on standardised weight‐bearing radiographs; HKA defined per Methods (positive = varus, negative = valgus).

Abbreviations: HKA, hip–knee–ankle; IQR, interquartile range; PSM, propensity‐score matched; SD, standard deviation.

* Continuous variables: mean ± SD if approximately normally distributed, otherwise median [IQR]; between‐group comparisons by Student's t‐test or Mann–Whitney *U* (two‐sided), as appropriate.

^a^ Effect size: Cohen's *d* (pooled SD).

### Surgical technique

All procedures were performed by the same senior surgical team from the Knee Surgery Unit, using a conventional (non‐robotic) technique, ensuring consistency in operative technique and decision‐making. The same implant model was used in all cases (Oxford® Phase 3 mobile‐bearing UKA system, Zimmer Biomet), and surgeries were conducted through a standardised medial parapatellar approach without patellar eversion. A limited arthrotomy was performed under spinal anaesthesia with the patient in a supine position and a proximal thigh tourniquet applied.

Bone resections were carried out using the dedicated instrumentation of the Oxford® Phase 3 mobile‐bearing UKA system (Zimmer Biomet), following the manufacturer's technique guide. The anterior cruciate ligament was preserved, and careful soft‐tissue balancing was undertaken to avoid overcorrection into valgus. Cemented fixation was employed in all cases.

Intraoperative and perioperative protocols included:

- Prophylactic antibiotics: cefazolin 2 g IV administered 30 minutes before incision (or vancomycin 1 g in beta‐lactam–allergic patients), with redosing after 4 h if necessary.
- Antifibrinolytic therapy: tranexamic acid 1 g IV at induction and 1 g at closure, unless contraindicated.
- DVT prophylaxis: low‐molecular‐weight heparin initiated 6–8 h after surgery, continued for 30 days.
- Pain management: a standardised multimodal analgesia protocol (paracetamol 1 g IV/8 h, dexketoprofen 50 mg IV/8 h, metamizole 1 g IV/8–12 h; tramadol 50–100 mg PO/IV as rescue medication when necessary).
- Rehabilitation: full weight‐bearing as tolerated beginning the same day of surgery. Physiotherapy focused on restoring full extension, active flexion, and early quadriceps activation.

Patients were routinely discharged between postoperative days two and three, with structured home‐based rehabilitation and instructions for progressive ambulation.

### Outcome measures and follow‐up

All postoperative clinical and functional outcomes were assessed and recorded by the same orthopedic surgeon throughout follow‐up, using structured templates and institutional protocols. Assessments were conducted at multiple time points:

- Preoperative baseline evaluation during the surgical indication visit.
- In‐hospital evaluation during the postoperative stay (pain, function, wound status).
- Routine follow‐up at 2 weeks, 1 month, 3 months, 6 months, and annually thereafter. For between‐group comparisons, patient‐reported outcome measures (PROMs: VAS, KSS, EQ‐5D and satisfaction) were analysed at the final follow‐up (≥12 months). We administered the New Knee Society Score (2011 update), using the validated Spanish version [3]. Health‐related quality of life was assessed with the EQ‐5D (Spanish version), using the EQ‐5D‐5L descriptive system developed in Spanish for Spain, with the corresponding Spanish valuation set [9].
- In a subset of patients with complete follow‐up, VAS and KSS were also collected at 1, 3 and 6 months to describe longitudinal evolution.

The following baseline variables were recorded:

- Age, sex and BMI.
- Laterality of the operated knee.
- Radiographic severity of osteoarthritis (Ahlbäck grade) in medial and patellofemoral compartments.
- HKA angle (coronal alignment).
- Comorbidities at the time of surgery, quantified by the Charlson Comorbidity Index.

**Primary outcomes** were:

- Postoperative pain (VAS).
- Functional outcome (KSS).
- Perioperative complications (local: aseptic loosening, infection, disease progression; systemic: e.g., myocardial infarction).

**Secondary outcomes** were:

- Range of motion (ROM), calculated as the difference between maximum active flexion and extension.
- Hospital for Special Surgery (HSS) score.
- Health‐related quality of life (EuroQol‐5D, EQ‐5D).
- Return to sport, assessed via standardised postoperative questionnaire at final follow‐up (months to resume self‐defined recreational activity: walking, swimming, cycling).
- Patient‐reported satisfaction: ‘satisfied’, ‘partially satisfied’, or ‘not satisfied’.
- Exploratory radiographic outcomes: femorotibial angle (pre‐/postoperative) and coronal/sagittal alignment of femoral and tibial components on standardised follow‐up radiographs.

Patients lost to follow‐up were excluded from all outcome analyses and were not mixed with completers, in order to avoid attrition bias.

### Statistical analysis

Analyses were conducted using SPSS version 26.0 (IBM Corp., Armonk, NY, USA). Continuous variables were summarised as mean ± standard deviation (SD) or as median (interquartile range) when non‐normally distributed. Categorical variables were reported as frequencies and percentages. Normality was assessed using the Shapiro–Wilk test. Between‐group comparisons were performed using Student's t test or Mann–Whitney *U* test for continuous variables, and *χ*² test or Fisher's exact test for categorical variables, as appropriate. Effect sizes were reported as Cohen's *d* for continuous variables and Cramér's V for categorical variables. No adjustment for multiplicity was applied given the exploratory nature of secondary analyses.

Propensity‐score matching was performed using binary logistic regression with the following pre‐specified covariates: age, body mass index, Charlson Comorbidity Index, baseline Knee Society Score (KSS), and preoperative visual analogue scale (VAS) pain. Nearest‐neighbour matching without replacement was applied with a caliper of 0.2 standard deviations of the logit of the propensity‐score. Covariate balance was confirmed using standard diagnostics. All between‐group inferences were conducted within the matched dataset.

## RESULTS

### Patient flow and matching

**Figure 1:**
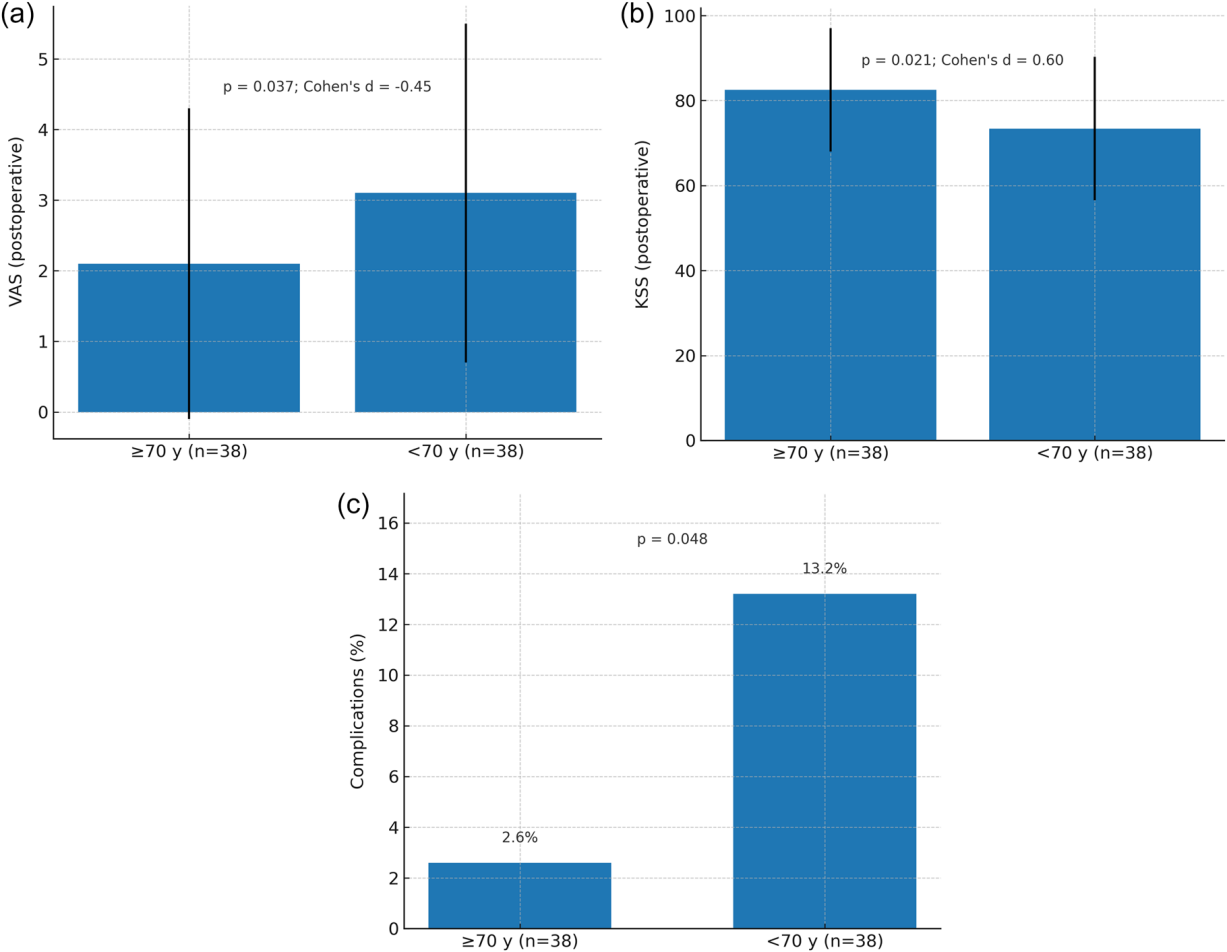
(a) Postoperative VAS in the propensity‐matched cohort. Bar chart showing mean ± SD postoperative VAS for ≥70 y and <70 y groups (PSM dataset only; *n* = 76). *p*‐value from Mann–Whitney *U* (two‐sided); Cohen's *d* reported. (b) Postoperative KSS in the propensity‐matched cohort. Bar chart showing mean ± SD postoperative KSS for ≥70 y and <70 y groups (PSM dataset only; *n* = 76). *p*‐value from Mann–Whitney *U* (two‐sided); Cohen's *d* reported. (c) Complication rates in the propensity‐matched cohort. Bar chart showing percentage of patients with ≥1 perioperative complication in each age group (PSM dataset only; *n* = 76). *p*‐value from chi‐square/Fisher's exact test (two‐sided). KSS, Knee Society Score; PSM, propensity‐score matched; SD, standard deviation; VAS, visual analogue scale.

All inferential analyses were confined to the propensity‐score–matched cohort, with no losses to follow‐up at ≥ 12 months. Figure 1a–c summarises the key outcomes.

### Baseline characteristics (PSM cohort)

After matching, groups were well balanced across matching covariates and coronal alignment descriptors (HKA). As summarised in Table 1, sex distribution, BMI, Charlson ≥1, Ahlbäck grades and HKA (°) were comparable between groups (all *p* ≥ 0.62).

### Primary and secondary outcomes (PSM cohort)

Elderly patients had lower postoperative pain (VAS 2.1 ± 2.2) than younger patients (3.1 ± 2.4; *p* = 0.037; Cohen's *d* = –0.45) while also experiencing fewer perioperative complications (2.6% vs. 13.2%; *p* = 0.048). They likewise showed higher postoperative function (KSS 82.5 ± 14.5 vs. 73.4 ± 16.9; *p* = 0.021; Cohen's *d* = 0.60) and reported a higher rate of satisfaction (97.4% vs. 84.2%; *p* = 0.049; Cramér's V = 0.25). Improvements from baseline were greater in elderly patients for both VAS (Δ –5.4 ± 2.5 vs. –4.3 ± 2.7; *p* = 0.019) and KSS (Δ 35.0 ± 13.8 vs. 27.0 ± 17.1; *p* = 0.026). Detailed estimates are provided in Table 2 and Figure 1a–c.

### Radiographic outcomes

Between‐group comparisons of postoperative femorotibial angle and component positioning (Table 3) showed small, non‐significant differences (all *p* ≥ 0.47). Coronal alignment is reported as HKA (°) only.

## DISCUSSION

In this study, patients aged ≥ 70 years undergoing medial UKA exhibited lower postoperative pain, higher functional scores, and fewer complications than younger patients. Specifically, within the matched cohort, elderly patients demonstrated lower VAS, higher KSS, and a lower complication rate, alongside a higher proportion of “satisfied” respondents at ≥12 months. These results support the view that age alone should not preclude UKA when contemporary selection criteria are met. These findings are concordant with recent syntheses indicating that chronological age does not increase failure risk and that outcomes in older cohorts are comparable to younger groups [11, 15] and with large comparative series in very elderly patients showing clinically meaningful gains and acceptable safety profiles [2, 5, 8, 21].

Therefore, in appropriately selected older patients, UKA can provide outcomes that are at least comparable—and in key domains, superior—to those in younger individuals. Several non‐exclusive mechanisms may contribute, including stricter indication in elderly candidates with truly isolated medial disease, lower activity demands that may reduce implant stresses, and standardised surgical pathways that limit variability and perioperative risk. These inferences are confined to the matched dataset; analyses of the unmatched cohort were not performed. Additionally, the matched sample size reflects the available cohort after applying strict eligibility criteria rather than a limitation of the matching method.

Clinically, these results imply that excluding candidates solely on the basis of age ≥70 is not supported. Indication should prioritise joint pathology (isolated compartment disease), coronal alignment, ligament competence, and realistic postoperative goals. Short‐term function after UKA has been reported to exceed that after TKA in appropriately indicated cases [18], and registry/epidemiologic evidence documents favourable utilisation and outcomes for UKA in contemporary practice [13, 16]. For older adults with limited physiological reserve, the combination of bone‐ and ligament‐sparing surgery, faster rehabilitation, and lower resource use further strengthens the rationale for UKA [12, 17].

Regarding selection and technique, consistent and reproducible criteria remain essential. Behavioural vignette data highlight ACL integrity as a key driver of surgeons' preference for UKA over TKA [14]. Technique also matters. Maintaining the native coronal phenotype and alignment has been associated with better functional results after ligament‐guided medial UKA [20]. The standardised approach used in this study—alignment‐preserving implantation and a single‐implant philosophy—likely contributed to the favourable recovery and low complication profile observed in elderly patients [6, 19]. In medial UKA, whole‐limb HKA is used descriptively rather than as a surgical target; preservation of the patient's native alignment and soft‐tissue balance is prioritised to avoid valgus over‐correction and lateral compartment overload. When radiographic variables guide indication or component positioning, reliability becomes important; arithmetic HKA measurement on long‐leg radiographs has demonstrated acceptable inter‐ and intra‐observer agreement [7].

From a comparative‐effectiveness and value standpoint, recent time‐driven activity‐based costing and value analyses indicate that, for isolated medial OA, UKA can reduce inpatient personnel time and total costs relative to TKA while maintaining patient‐level value [12, 17]. These considerations are particularly relevant in older populations where deconditioning and comorbidity magnify the impact of length of stay and complication avoidance. Care pathways that reserve TKA for multi‐compartment arthritis but offer UKA to elderly candidates with strict medial disease may thus improve both outcomes and efficiency [12, 17, 18].

With respect to external validity and service delivery, generalisability depends on context. Outcomes in UKA are sensitive to surgeon and institutional experience; database analyses associate higher surgeon volume with improved survivorship [1]. The present single‐centre study reflects a mature protocol with consistent indication, implant, and perioperative care; similar outcomes should be expected where comparable expertise and pathways exist. Registry‐based findings further suggest that, at the population level, UKA performance continues to improve with contemporary technique and selection [16].

Strengths of this study include standardised implant and perioperative protocols, consistent indication and care pathways at a single centre, and restriction of inferential analyses to a matched cohort to minimise confounding. Limitations include the retrospective single‐centre design; a modest matched sample that limits precision for rare events; the study was not powered to detect very low‐frequency adverse events, and larger multicenter series are warranted for robust safety estimates; minimum follow‐up that precludes long‐term survivorship inferences; postoperative outcomes assessed by a single evaluator, which precludes inter‐rater reliability assessment; lack of formal inter‐ and intra‐observer reliability testing for all radiographic measures; and no multiplicity adjustment for secondary endpoints. Interpretation of non‐significant results as 'trends' was avoided.

Future work should prioritise prospective multicenter cohorts with longer follow‐up, standardised radiographic reliability assessments, and serial PROMs aligned with current reporting frameworks, as well as health‐economic endpoints (e.g., time‐driven costing, readmission‐adjusted value) and decision‐analytic models comparing UKA and TKA pathways in septuagenarians and octogenarians [12, 16, 17, 18].

Taken together, these findings indicate that chronological age ≥ 70 years, by itself, should not determine candidacy for medial UKA when contemporary selection criteria and experienced care pathways are applied.

## CONCLUSIONS

In appropriately selected patients aged ≥70 years, medial unicompartmental knee arthroplasty yields clinical outcomes and satisfaction that are at least comparable—and in several measures superior—to those of younger patients at a minimum of 12 months' follow‐up; therefore, chronological age alone should not preclude indication for medial UKA.

## AUTHOR CONTRIBUTIONS

Miguel Ángel Muñoz‐Sánchez, Ana Martínez‐Crespo, and Juan Miguel Gómez‐Palomo contributed equally and substantially to the conception and design of the study, data analysis, drafting of the manuscript, and critical revisions. Juan Miguel Gómez‐Palomo also played a key role in the supervision and coordination of the entire research and writing process. Carmen Tara‐Abad, María Ríos‐Morón, and Elvira Montañez‐Heredia contributed to data collection, literature review, and assisted in the final review of the manuscript, with a secondary but valuable role. All authors read and approved the final version of the manuscript and agree to be accountable for its contents.

## CONFLICT OF INTEREST STATEMENT

The authors declare no conflicts of interest.

## ETHICS STATEMENT

Approved by the Research Ethics Committee of the Province of Málaga (SICEIA‐2024‐001425). Informed consent was obtained from all participants included in the study.

## DECLARATION

We used ChatGPT (OpenAI) solely for language polishing (grammar, syntax, and minor phrasing). No AI was used for literature search, data collection/analysis, interpretation of results, or creation of scientific content/images, and no identifiable patient data or unpublished datasets were entered. All authors reviewed and approved the manuscript, take full responsibility for its content, and AI tools are not listed as authors.

## Data Availability

Data available on reasonable request from the corresponding author.
